# Fe deficiency differentially affects the vacuolar proton pumps in cucumber and soybean roots

**DOI:** 10.3389/fpls.2013.00326

**Published:** 2013-08-27

**Authors:** Marta Dell’Orto, Patrizia De Nisi, Gianpiero Vigani, Graziano Zocchi

**Affiliations:** Dipartimento di Scienze Agrarie, Alimentari e Ambientali, Università degli Studi di MilanoMilano, Italy

**Keywords:** V-ATPase, V-PPase, Fe deficiency, cucumber, soybean

## Abstract

Iron uptake in dicots depends on their ability to induce a set of responses in root cells including rhizosphere acidification through H^+^ extrusion and apoplastic Fe(III) reduction by Fe(III)-chelate reductase. These responses must be sustained by metabolic rearrangements aimed at providing the required NAD(P)H, ATP and H^+^. Previous results in Fe-deficient cucumber roots showed that high H^+^ extrusion is accompanied by increased phosphoenolpyruvate carboxylase (PEPC) activity, involved in the cytosol pH-stat; moreover ^31^P-NMR analysis revealed increased vacuolar pH and decreased vacuolar [inorganic phosphate (Pi)]. The opposite was found in soybean: low rhizosphere acidification, decreased PEPC activity, vacuole acidification, and increased vacuolar [Pi]. These findings, highlighting a different impact of the Fe deficiency responses on cytosolic pH in the two species, lead to hypothesize different roles for H^+^ and Pi movements across the tonoplast in pH homeostasis. The role of vacuole in cytosolic pH-stat involves the vacuolar H^+^-ATPase (V-ATPase) and vacuolar H^+^-pyrophosphatase (V-PPase) activities, which generating the ΔpH and ΔΨ, mediate the transport of solutes, among which Pi, across the tonoplast. Fluxes of Pi itself in its two ionic forms, H_2_PO_4_^-^ predominating in the vacuole and HPO_4_^2-^ in the cytosol, may be involved in pH homeostasis owing to its pH-dependent protonation/deprotonation reactions. Tonoplast enriched fractions were obtained from cucumber and soybean roots grown with or without Fe. Both V-ATPase and V-PPase activities were analyzed and the enrichment and localization of the corresponding proteins in root tissues were determined by Western blot and immunolocalization. V-ATPase did not change its activity and expression level in response to Fe starvation in both species. V-PPase showed a different behavior: in cucumber roots its activity and abundance were decreased, while in Fe-deficient soybean roots they were increased. The distinct role of the two H^+^ pumps in Pi fluxes between cytoplasm and vacuole in Fe-deficient cucumber and soybean root cells is discussed.

## INTRODUCTION

Among Strategy I plants (dicots and non-graminaceous monocots) there is an inter- and intra-specific variability in susceptibility to lime-induced Fe deficiency, despite a similar demand for Fe. This variability is strongly related to the ability of plants to enhance the activities involved in the so-called reduction based Strategy I (Marschner et al., 1986; Schmidt, 2006; Kim and Guerinot, 2007; Ivanov et al., 2012), residing at the root cell plasma membrane (PM), which consists of three main steps: (1) acidification of the root apoplast and rhizosphere, mainly due to the enhanced H^+^ extrusion driven by the plasma membrane H^+^-ATPase (PM-ATPase); (2) reduction of the extracellular Fe^3+^ to Fe^2+^, which is the only form transported into the root by these species, by means of Fe(III)-chelate reductase (FC-R) which utilizes NAD(P)H as reducing substrate; (3) Fe^2+^ transport into the root symplast by IRT1, a member of the ZIP family.

The variability found among plants in the ability to induce these activities under Fe deficiency is particularly wide for H^+^ extrusion (Schmidt, 1999) rather than for FC-R, since Fe(III) reduction is an obligatory step in Fe acquisition by Strategy I plants; in fact, differences were found among differently tolerant species also in the ability to modify the metabolism in order to provide NAD(P)H, ATP and H^+^ necessary to sustain the reduction-based response (Zocchi, 2006).

Moreover, a wide variability has been also found among dicots in the production and secretion of compounds facilitating the uptake of Fe, such as flavins and phenolics (Rodríguez-Celma et al., 2013).

In Fe-deficient cucumber plants the high induction of the Strategy I responses is concomitant with the increase in carbohydrate catabolism, through the up-regulation of glycolysis and oxidative pentose phosphate pathway (Rabotti et al., 1995; Espen et al., 2000), providing ATP and reducing equivalents. Moreover, in such conditions, phosphoenolpyruvate carboxylase (PEPC) activity results to be enhanced even more than the primary responses (De Nisi and Zocchi, 2000), leading to PEP consumption (with further increase in the glycolysis rate) and synthesis of organic acids, which are protogenic. Indeed, PEPC activity has been recognized to play a fundamental role in balancing cytoplasmic pH (Davies, 1973), which, in Fe-deficient cucumber roots, is subject to alkalization due to the exceptionally high rate of H^+^ extrusion. Intriguingly, a strong PEPC induction upon Fe deficiency was found in plants with high H^+^ extrusion activity such as sugar beet (López-Millán et al., 2000), bean (Bienfait et al., 1989), *Capsicum annuum* (Landsberg, 1986). On the contrary, in plants which do not induce H^+^-ATPase activity under Fe starvation, or which induce it at a low rate such as *Medicago ciliaris* (M’sehli et al., 2009a,b) or soybean (Zocchi et al., 2007), PEPC activity and expression level only are weakly or not induced.

Thus, comparing the responses to Fe deficiency in two dicotyledonous plants, cucumber, and soybean, it arises that their different ability to induce the Strategy I responses and in particular apoplast acidification, is accompanied by different degree in the activation of the carbohydrate catabolism, especially in the induction of PEPC (De Nisi and Zocchi, 2000; Zocchi et al., 2007). Accordingly, data from *in vivo* ^31^P-NMR studies conducted on Fe deficient roots of these two species (Espen et al., 2000; Zocchi et al., 2007) showed some interesting differences: (a) the cytoplasm pH was kept almost constant in root cells of both species, suggesting that efficient pH-stat mechanisms are functioning; conversely, the vacuolar pH increased in cucumber, characterized by active H^+^ extrusion to the apoplast, while it was lowered in soybean roots under Fe deficiency; this is consistent with the role of vacuole in cytosolic pH homeostasis under various stresses (Martinoia et al., 2007); (b) moreover, while in Fe-deficient cucumber roots a dramatic fall in the vacuolar inorganic phosphate (Pi) concentration occurred, soybean showed a strong increase of the Pi concentration in the vacuole. These findings led Zocchi et al. (2007) to hypothesize that, besides the already established role of PM-ATPase and PEPC in the pH-stat mechanism under Fe deficiency (Zocchi, 2006 and references therein), also the movements of Pi in its two inorganic forms, H_2_PO_4_^-^ predominating in the vacuole and HPO_4_^2-^ in the cytosol, across the tonoplast could cooperate in balancing the cytosolic pH, thanks to the buffering activity exerted by its pH-dependent protonation/deprotonation reactions. Little is known about Pi transport across the tonoplast, as no carriers nor channels have been identified so far. What is known is that its entrance into the vacuole has been demonstrated to be driven by the H^+^ gradient generated across the tonoplast by the two vacuolar proton pumps (Massonneau et al., 2000; Ohnishi et al., 2007): the vacuolar H^+^-ATPase (V-ATPase, EC 3.6.3.14) and the vacuolar H^+^-pyrophosphatase (V-PPase, EC 3.6.1.1), which actively transport H^+^ inside the vacuolar lumen hydrolyzing ATP and inorganic pyrophosphate (PPi), respectively, thus generating the electrochemical potential difference (ΔpH and ΔΨ) which mediates the secondary active transport of solutes (among which Pi) across the tonoplast (Maeshima, 2001; Hedrich and Marten, 2006; Schumacher, 2006).

Thus, the activity and expression of V-ATPase and V-PPase are expected to be differently regulated in cucumber and soybean in response to Fe deficiency. For this reason, the aim of this work was to characterize how the activities of the vacuolar proton pumps change in cucumber and soybean roots grown under Fe deficiency.

## MATERIALS AND METHODS

### PLANT MATERIAL AND GROWTH CONDITIONS

Seeds of cucumber (*Cucumis sativus* L., cv. Marketmore 76) and soybean (*Glycine max *L. cv. Elvir from Pioneer, Italy) were sown in wet agriperlite and allowed to germinate in the dark at 26°C for 3 days (cucumber) or 18°C for 6 days (soybean). Seedlings were transferred to a nutrient solution with the following composition: 2 mM Ca(NO)_3_, 0.75 mM K_2_SO_4_, 0.65 mM MgSO_4_, 0.5 mM KH_2_PO_4_, 10 μM H_3_BO_3_, 1 μM MnSO_4_, 0.5 μM ZnSO_4_, 0.05 μM (NH_4_)Mo_7_O_24_, and 0.1 mM Fe-EDTA (when added). The pH was brought to 6.0–6.2 with NaOH. Plants in hydroponic cultures were maintained in a growth chamber with a day/night regime of 16/8 h at 24/18°C and a PPFD of 200 μmol m^-2^ s^-1^. The nutrient solution was changed weekly.

### ISOLATION OF TONOPLAST-ENRICHED VESICLES

Vacuolar membrane vesicles were isolated from roots of plants grown for 8 days (cucumber) and 11 days (soybean) in the presence or in the absence of Fe, according to Rea and Poole (1985) with some modifications. About 25 g of roots were homogenized using mortar and pestle in 4 mL/g (fresh weight) of a buffer containing 250 mM sorbitol, 25 mM Tris-Mes pH 7.4, and 5 mM Na-EDTA. Just prior to use, 1 mM DTT and 2 mM PMSF were added to the buffer. The homogenate was filtered through four layers of gauze and centrifuged at 12,000 *g* for 15 min. The supernatant was then centrifuged at 80,000 *g* for 30 min. The pellet was resuspended in 3 mL of a resuspension medium (RM) containing 1.1 M glycerol, 2.5 mM Tris-Mes pH 7.4, 5 mM Na-EDTA, 1 mM DTT, and 0.1 mM PMSF and layered over a 10/23% discontinuous sucrose gradient prepared in RM. After centrifugation at 80,000 *g* (r_max_) for 2 h in a swinging bucket rotor (SW40), vesicles sedimented at the interface between 10 and 23% sucrose were collected, diluted with three volumes of RM and centrifuged at 80,000 *g* for 30 min. The pellet was finally resuspended in about 150 μL of RM. The vesicles were either used immediately or frozen under liquid N_2_ and stored at -80°C until use. Protein concentration was determined by the Bradford method using BSA as the standard (Bradford, 1976).

### MEASUREMENT OF H^+^-ATPase AND H^+^-PPase ACTIVITIES

The activity of the V-ATPase was measured as the rate of ADP-dependent NADH oxidation in a coupled lactate dehydrogenase-pyruvate kinase reaction and ATP-regenerating system at 25°C according to Ward and Sze (1992) with modifications. The reaction mixture (1 mL) contained 25 mM MOPS-BTP buffer pH 7.0, 50 mM KCl, 250 mM sucrose, 3 mM ATP, 1 mM PEP, 0.25 mM NADH, 4.5 mM MgSO_4_, 0.015% Lubrol, 6 U/mL pyruvate kinase and 12 U/mL lactate dehydrogenase, 20 μg membrane protein. Sodium molybdate 0.1 mM was added to the reaction medium to inhibit acid phosphatase activity. The rate of NADH oxidation was measured as the decrease in A_340_ with time. The assay was preformed in the presence and in the absence of 50 mM K-nitrate (a specific inhibitor of the V-type ATPase) and the difference between these two activities was attributed to the vacuolar ATPase. Analog results were obtained using bafilomycin as V-ATPase inhibitor. The degree of purity of the tonoplast membrane preparations was also assessed by measuring marker enzymes for mitochondria (azide-sensitive H^+^-ATPase) and PM (vanadate-sensitive H^+^-ATPase) contamination according to Rabotti and Zocchi (1994): around 60% of the total activity was nitrate-sensitive (tonoplast), the remaining 40% was almost totally vanadate-sensitive activity, while the azide-sensitive activity was negligible.

The activity of the V-PPase was measured as the rate of liberation of Pi from PPi in a reaction volume of 250 μl by the Ames (1966) method. According to Rea and Poole (1985) with some modifications, the assay medium consisted of 15 μg membrane protein, 40 mM Tris-Mes pH 8.0, 50 mM KCl, 0.1% Lubrol, 3 mM Na-PPi and 3 mM MgSO_4_, 0.1 mM Na_2_MoO_4_ (to inhibit acid phosphatase activity). PPase activity was calculated as half the rate of Pi liberation (1 mol of PPi = 2 mol of Pi).

### WESTERN BLOTTING ANALYSIS

Tonoplast enriched fractions extracted from roots of plants grown in the presence and in the absence of Fe were loaded on a discontinuous SDS-polyacrylamide gel (3.75% [w/v] acrylamide stacking gel, and 10% [w/v] acrylamide separating gel). For each sample 10 μg of total protein were loaded.

After SDS-PAGE, the electrophoretic transfer to nitrocellulose membrane filters (Sigma) was performed using a semi-dry blotting system in 10 mM cyclohexylamino-1-propane sulphonic acid (pH 11.0 with NaOH) and 10%(v/v) methanol for 1.5 h at room temperature at 0.8 mA cm^-2^. After blotting, the membrane was incubated for 1 h in TBS-T buffer (Tris Buffered Saline, 0.1% Tween-20, 5% commercial dried skimmed milk). Polyclonal antibodies raised against a 100 kDa peptide (ELVEINANNDKLQRSYNELC) corresponding to the V-ATPase subunit a and a 73 kDa peptide (CDLVGKIERNIPEDDPRN) corresponding to the V-PPase (Maeshima and Yoshida, 1989; Maeshima, 2001; Kobae et al., 2004) were used. The incubation in primary antibody, diluted 1:1500 in TBS, was carried out for 2 h at room temperature. After rinsing with TBS-T, membranes were incubated at room temperature for 2 h with a 1:10000 diluted secondary antibody (alkaline phosphatase-conjugated anti-rabbit IgG, Sigma). After rinsing in TBS-T membranes were incubated in 5-bromo-4-chloro-3-indolyl phosphate and nitroblue tetrazolium (FAST BCIP/NBT, Sigma).

### IMMUNOLOCALIZATION OF VACUOLAR PROTON PUMPS

Apical segments from roots of both 8-day-old cucumber and 11-day-old soybean plants grown in the presence and in the absence of Fe were fixed at 4°C in 100 mM sodium phosphate buffer (pH 7.0) containing 4% paraformaldehyde (w/v), then dehydrated through an ethanol-tertiary butanol series and embedded in paraffin (Paraplast plus, Sigma) as described by Dell’Orto et al. (2002). Serial sections of 5 μm were cut with a microtome up to a distance from the tip of about 1500 μm. Sections were mounted on polylysine-treated slides, deparaffinized in xylene and rehydrated through an ethanol series.

Immunological detection was performed as in Dell’Orto et al. (2002) with some modifications. Sections were incubated for 30 min at room temperature in 3% H_2_O_2_, then blocked for 1 h in TBS (150 mM NaCl, 25 mM Tris-HCl, pH 7.6) with 2% BSA (w/v). Sections were incubated overnight at 4°C in the same polyclonal antibodies used for Western blot after dilution 1:200 (anti-V-ATPase) and 1:300 (anti-V-PPase) in TBS with 0.5% BSA. After 3 × 5 min washes in TBS, sections were incubated 2 h at room temperature with a biotinylated secondary antibody (anti-rabbit IgG biotin conjugate developed against goat, Sigma) diluted 1:200 in TBS with 0.5% BSA. After 3 × 5 min washes in TBS sections were incubated 30 min in extravidin-peroxidase (ExtrAvidin Peroxidase Staining Kit, Sigma) diluted 1:20. After 3 × 5 min washes in TBS sections were incubated 3–5 min in 0.05 M acetate buffer pH 5.0 containing 5% dimethylformamide, 0.04% 3-amino-9-ethylcarbazole (AEC) and 0.015% H_2_O_2_ and finally washed in distilled water.

## RESULTS

Cucumber and soybean plants grown in Fe-deprived nutrient solution for 8 and 11 days, respectively, showed highly chlorotic leaves and the typical Fe deficiency responses already described in previous works at the root level (Rabotti et al., 1995; Espen et al., 2000; Zocchi et al., 2007): in cucumber, a strong increase in both Fe reduction and H^+^ extrusion, by induction of FC-R and PM-ATPase activities respectively, enhanced activity of glycolytic enzymes and PEPC; in soybean, increased Fe reduction activity but weak acidification of the nutrient solution, no induction of the PM-ATPase activity, only slight increase of glycolytic enzymes and decrease of PEPC activity (data not shown).

For this reason, in order to investigate the role played by the two vacuolar H^+^ pumps in the pH-stat mechanism under Fe deficiency, vacuolar membrane vesicles were isolated from roots of both 8-day-old cucumber and 11-day-old soybean plants grown in the presence and in the absence of Fe. The V-ATPase activity, determined as the difference between ATP hydrolysis in the presence and in the absence of 50 mM K-nitrate (nitrate-sensitive ATPase) was around 60% of the total ATPase activity for both cucumber and soybean preparations and is shown in **Table 1**. Both in cucumber and in soybean roots this activity is diminished under Fe deficiency. The V-PPase (**Table 2**) shows a similar behavior as the V-ATPase in Fe-deficient cucumber roots, lowering its phosphohydrolytic activity and thus contributing to the vacuole alkalization. On the contrary, in Fe-deficient soybean roots this activity is kept at the same level of the control or even slightly increased.

**Table 1:** Nitrate-sensitive H^+^-ATPase activity determined on tonoplast enriched fractions isolated from roots of 8-day-old cucumber and 11-day-old soybean plants grown in the presence (control) and in the absence of iron (-Fe).

ATPase activity (nmol NADH mg^-1^ prot min^-1^)			
	Control	-Fe	%
**Cucumber**
	453 ± 31	290 ± 18	-36
**Soybean**
	168 ± 12	94 ± 8	-44

*Results are the mean values (±SE) of four experiments.*

**Table 2:** Vacuolar H^+^-PPase activity determined on tonoplast enriched fractions isolated from roots of 8-day-old cucumber and 11-day-old soybean plants grown in the presence (control) and in the absence of iron (-Fe).

PPase activity (nmol PPi mg^-1^ prot min^-1^)			
	Control	-Fe	%
**Cucumber**
	215 ± 15	129 ± 11	-40
**Soybean**
	179 ± 15	194 ± 11	+8

*Results are the mean values (±SE) of four experiments.*

**FIGURE 1:**
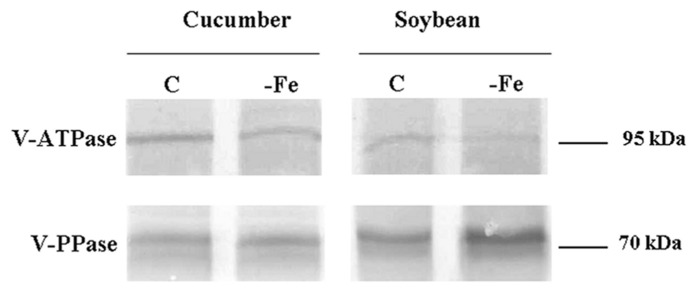
**Western blot analysis of proteins (10 μL per lane) present in tonoplast-enriched membrane vesicle fractions isolated from cucumber (left) and soybean (right) roots grown in the presence (C) and in the absence (-Fe) of Fe.** Immunostaining was performed using polyclonal antibodies raised against the V-ATPase a subunit and the V-PPase polypeptide respectively.

In order to verify whether the H^+^ pump activities are regulated, under Fe deficiency, by decreasing their synthesis, Western bolt analysis was performed on tonoplast enriched fractions using two polyclonal antibodies raised against the V- PPase polypeptide and the a subunit of the V-ATPase, respectively. For what concerns the V-ATPase (**Figure 1**) the antibody recognized a polypeptide with a molecular mass of about 95 kDa. The results are consistent with the measured activities, showing a decrease in the protein level in Fe-deficient roots of both cucumber and soybean. Also the V-PPase polypeptide level reflects the activity changes found under Fe deficiency, showing a decreased level of protein in cucumber roots and an accumulation in soybean (**Figure 1**).

**FIGURE 2:**
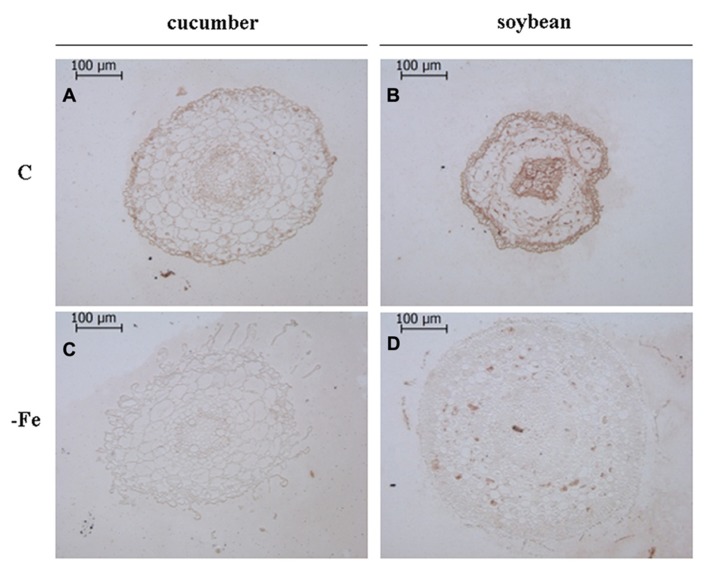
**Immunolocalization ofV-ATPase in cross sections of 8-day-old cucumber and 11-day-old soybean roots.** C: control (Fe-sufficient) cucumber **(A)** and soybean **(B)** root sections; -Fe: Fe-deficient cucumber **(C)** and soybean **(D)** root sections. Root sections were cut at 500–900 μm from the tips, corresponding to the elongation zone. The sections treated without the primary antibody appeared not stained, indicating the absence of any aspecific reaction (data not shown).

**FIGURE 3:**
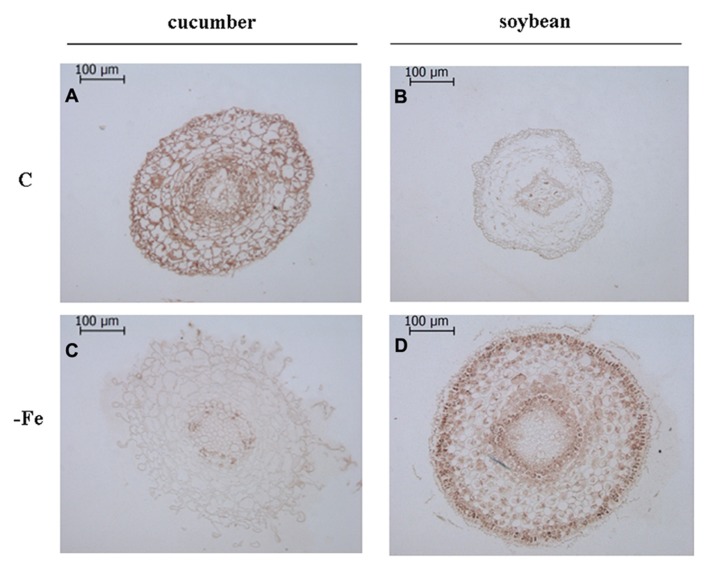
**Immunolocalization ofV-PPase in cross sections of 8-day-old cucumber and 11-day-old soybean roots.** C: control (Fe-sufficient) cucumber **(A)** and soybean **(B)** root sections; -Fe: Fe-deficient cucumber **(C)** and soybean **(D)** root sections. Root sections were cut at 500–900 μm from the tips, corresponding to the elongation zone. The sections treated without the primary antibody appeared not stained, indicating the absence of any aspecific reaction (data not shown).

Moreover, by using the same antibodies, an *in situ* immunological detection of V-ATPase and V-PPase proteins has been performed on subapical root sections of cucumber and soybean plants grown in the presence and in the absence of Fe (**Figures 2** and **3**) in order to localize this response at the histological level. Root sections in **Figures 2** and **3** were cut at 500–900 μm from the tips, corresponding to the elongation zone. Root sections treated without the primary antibody appeared not stained, indicating that aspecific reactions attributable to the secondary antibody or to extravidin-peroxidase complex did not occur (data not shown).

Overall, it is evident that growth in the absence of Fe leads to the proliferation of root hairs in cucumber (**Figures 2A,C** and **3A,C**) and to increased root diameter in soybean (**Figures 2B,D** and **3B,D**). This is in agreement with the well documented occurrence of root morphological modifications such as swollen root tips and root hairs formation in several Fe-deficient Strategy I plants (Schmidt, 1999; Dell’Orto et al., 2002). Under control growth conditions, the V-ATPase accumulated mainly in the external cell layers of root sections from both cucumber and soybean plants (**Figures 2A,B**). When grown in the absence of Fe, both cucumber and soybean root sections show a markedly reduced level of the V-ATPase (**Figures 2C,D**). The V-PPase protein is widely accumulated in root sections from the control cucumber plants (**Figure 3A**) staining all cortex layers, while under Fe deficiency a strong decrease in V-PPase protein expression occurs (**Figure 3C**), even more drastic than what detected by immunoblot analysis; soybean plants show a lower steady-state level of the V-PPase protein in the same root zone, but under Fe deficiency the PPase is strongly intensified, localizing particularly at the external cell layers and at the endodermis, suggesting an increase expression of the protein, in agreement with the Western blot result.

## DISCUSSION

It is becoming more and more evident that, beyond the obligatory step of Fe reduction, the response efficiency in adapting to Fe low availability in Strategy I plants resides on other mechanisms which can be differently regulated in different genotypes. In particular, the highly differentiated aptitude to acidify the apoplast and the rhizosphere, by inducing the PM-ATPase activity, appears to explain the different tolerance exhibited by different genotypes to calcareous soils (Schmidt, 1999; Ksouri et al., 2006; M’sehli et al., 2009b).

By comparing the response to Fe deficiency in two Strategy I species, cucumber and soybean, it emerged that the different aptitudes to induce the PM-ATPase are accompanied by other differences, related to the root cell vacuolar composition: in cucumber roots, in which PM-ATPase and PEPC activities are strongly induced, the vacuole undergoes an alkalization and a depletion of Pi, while in soybean, in which PM-ATPase and PEPC are weakly and/or not induced, the vacuolar pH is decreased and the Pi concentration increased (Espen et al., 2000; Zocchi et al., 2007). Thus, besides the already established role of PM-ATPase and PEPC in the pH-stat mechanism under Fe deficiency (Zocchi, 2006 and references therein), also the movements of Pi across the tonoplast could cooperate in balancing the cytosolic pH, thanks to the buffering activity exerted by its pH-dependent protonation/deprotonation reactions. So, to explain the different movements of Pi in soybean and cucumber, we hypothesized that in Fe-deficient soybean roots the net influx of Pi toward the vacuole would be sustained by the increased H^+^ pumping activity of V-ATPase and/or V-PPase. On the contrary, in Fe-deficient cucumber roots we expected a decreased activity of the vacuolar proton pumps, perhaps to counterbalance the strong H^+^ consumption by PM-ATPase and leading to a release of H_2_PO^-^ which, by deprotonation in the cytosol, would cooperate in buffering the cytosolic pH.

**FIGURE 4:**
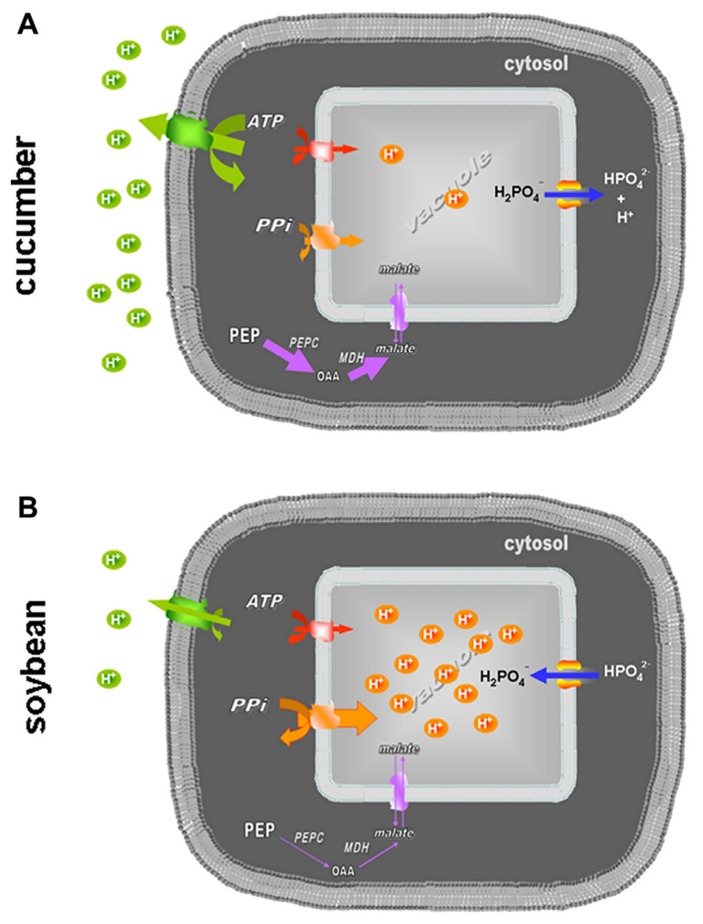
**Representation of the different roles of the vacuolar proton pumps and Pi movements across the tonoplast in cucumber (A) and soybean (B) roots under Fe deficiency.** The activities of the PM-ATPase, V-ATPase, V-PPase, and PEPC are visualized as differently marked lines; the net Pi fluxes are described through the arrow orientation.

To test these hypotheses, tonoplast-enriched fractions were extracted from roots of cucumber and soybean plants grown in the presence and in the absence of Fe. In cucumber roots, Fe starvation induced a reduced activity of both the nitrate-sensitive ATPase and the PPase (**Tables 1** and **2**), as expected considering the high ATP and H^+^ consumption by PM-ATPase and the increased vacuolar pH detected under these conditions (Rabotti and Zocchi, 1994; Espen et al., 2000). This finding fits well with the scenario depicted in **Figure 4A**, resuming all the data available so far for cucumber: the increased PM-ATPase activity induces the alkalinization of the cytoplasm pH which, in turn, activates the PEPC (Zocchi, 2006). Moreover, the high need for H^+^ is in part fulfilled also by the increased glycolysis rate; in part by the lower H^+^ transport into the vacuole due to the reduced vacuolar pump activity, in part, as a consequence, by the net efflux of H_2_PO_3_^-^, which, at the cytosolic pH is subject to dissociation into H^+^ and HPO_3_^2-^ (the pKa being 6.8).

For Fe-deficient soybean the proposed scenario is different, as shown in **Figure 4B**. In soybean roots the activity of the vacuolar pumps are differently affected by Fe deficiency: unexpectedly the V-ATPase activity is lowered more or less at the same degree as in cucumber, despite the low activity of the antagonistic PM-ATPase, showing to be not responsible for the acidification of the vacuole detected by Zocchi et al. (2007). On the contrary, the slight increase of the V-PPase activity found in Fe-deficient soybean roots, besides fitting well with the already mentioned vacuole acidification, is in agreement with several works reporting the increase of such activity under mineral deficiencies and various other stresses which affect the ATP synthesis rate (Maeshima, 2000 and references therein). In fact PPi, despite its high-energy phosphoanhydride bond, is indeed a low-cost substrate, generated as a by-product of several metabolic processes characteristic of actively growing cells, such as the synthesis of proteins, nucleic acid, and cellulose (Maeshima, 2000; Gaxiola et al., 2007). Thus, the ability of soybean roots to induce the V-PPase rather than the V-ATPase activity in response to a stress condition, which deeply affects the mitochondrial efficiency and thus the ATP supply (Vigani, 2012), could be interpreted as a metabolic plasticity leading to an alternative adaptive mechanism. In soybean roots, differently to what happens in cucumber the glycolysis rate does not increase significantly (Zocchi et al., 2007).

Protein amounts of V-ATPase subunit a and V-PPase were slightly reduced in the tonoplast fractions extracted from Fe-deficient cucumber roots (**Figure 1**). Thus, the observed reduction in the activity of both tonoplast H^+^ pumps can be, at least in part, attributed to changes in the expression of the encoding genes or in the amounts of proteins. As well, the amount of V-ATPase subunit a detected by Western blot in Fe-deficient soybean roots was lowered (**Figure 1**), consistently with the specific activity, while the amount of V-PPase (**Figure 1**), whose activity was found to be increased by Fe starvation, was higher. This is not an obvious result since it has been found, especially for the V-PPase, that the activity changes in response to nutritional stresses are due to post-translational modulation rather than to increased amount of the proteins (Maeshima, 2000; Ohnishi et al., 2007; Kabała and Janicka-Russak, 2011).

Several post-translational regulation mechanisms have been proposed both for V-ATPase and for V-PPase in plants: one of the possible mechanisms involved in the regulation of V-ATPase is reversible phosphorylation (Liu et al., 2004; McCubbin et al., 2004; Hong-Hermesdorf et al., 2006); moreover, a phosphorylation-dependent specific interaction with 14-3-3 protein has been found to be involved (Klychnikov et al., 2007). Since a putative 14-3-3 interaction motif has been identified also in V-PPase from *Vitis vinifera*, the existence of a coordinated regulation of the three H^+^ pumps involved in pH-homeostasis in the cytosol can be postulated (Gaxiola et al., 2007). The post-translational regulation of plant V-ATPase has been reviewed by Ratajczak (2000).

It has been found that in young growing tissues in which the anabolism is high, such as for instance seedling hypocotyls, the V-PPase activity determined on vacuolar membrane fraction was higher respect to the V-ATPase activity, due to a higher content of the former enzyme, this being consistent with the availability of large amounts of PPi in such tissues. On the contrary, in mature cells, in which the anabolism decreases leading to a lower PPi availability, V-PPase activity has been found to be lower than that of V-ATPase (Maeshima, 2000 and references therein). In this work the immunohistochemical technique allowed us to localize the changes in protein amounts in the root portion in which the response to Fe deficiency actually occurs, i.e., the sub apical region, corresponding to the elongation zone, characterized by actively growing tissues and thus by a high anabolic activity. Under Fe-sufficient conditions the relative enrichment of the V-PPase protein respect to the V-ATPase could be seen only in cucumber roots; while in soybean roots the V-ATPase seems to be more expressed than the V-PPase. Moreover, the differences found between Fe-sufficient and Fe-deficient roots in the expression level of the two proteins appear to be stronger than what evidenced by western blot. This could be probably explained by the fact that the tonoplast enriched fraction was obtained from the whole root system, leading to a dilution effect in the control samples, since the two proton pumps, but especially the V-PPase, are particularly expressed in the actively growing tissues (Nakanishi and Maeshima, 1998; Maeshima, 2000; Gaxiola et al., 2007) as the root meristems and elongation zones are. With this regard, the immunohistochemical approach seems provide better assessment, since it allows to detect the expression of the two proton pumps in a more limited region, in which the Strategy I responses actually take place (Dell’Orto et al., 2002; Schmidt et al., 2003).

Overall, the results obtained in this work, together with those previously reported by Espen et al. (2000) and Zocchi et al. (2007) suggest that cucumber and soybean, although both adopting the Strategy I for Fe uptake, follow different models for what concerns the metabolic adaptations needed to sustain the response to Fe starvation. In particular these two species resulted to differently manage the cellular pH homeostasis by differently modulating the V-PPase and, as a consequence, by differently regulating the Pi fluxes across the tonoplast.

It is still to be elucidated how the vacuolar proton pumps regulation is coordinated with that of the other activities involved in the pH-stat of the cell, i.e., the PM-ATPase and the PEPC. Moreover, also the fluxes of malate produced by PEPC across the tonoplast could be involved in the cytosolic pH homeostasis as proposed by Martinoia et al. (2007) and by **Figure 4**, since under Fe deficiency the malate concentration is strongly increased in several dicotyledonous species, among which cucumber (Rabotti et al., 1995). Future work should then be addressed to elucidate the role of malate and the regulation of the vacuolar malate transporter, since acidification of the plant cytosol has been found to stimulate the expression of the gene encoding for it in *Arabidopsis* (*At*tDT; Hurth et al., 2005).

## Conflict of Interest Statement

The authors declare that the research was conducted in the absence of any commercial or financial relationships that could be construed as a potential conflict of interest.
